# Safety and outcome of gastrostomy tube placement in patients with Loeys-Dietz syndrome

**DOI:** 10.1186/s12876-020-01213-2

**Published:** 2020-03-12

**Authors:** Pamela A. Frischmeyer-Guerrerio, Gretchen MacCarrick, Harry C. Dietz, F. Dylan Stewart, Anthony L. Guerrerio

**Affiliations:** 1 The Laboratory of Allergic Diseases, National Institutes of Allergy and Infectious Diseases, Bethesda, MD USA; 2 McKusick-Nathans Institute of Genetic Medicine, Johns Hopkins University School of Medicine, Baltimore, MD USA; 3 Howard Hughes Institute, Chevy Chase, MD USA; 4 Department of Surgery, Westchester Medical Center, Section of Pediatric Surgery, Maria Fareri Children’s Hospital, Valhalla, NY USA; 5 Department of Pediatrics, Division of Pediatric Gastroenterology, Hepatology and Nutrition, Johns Hopkins University School of Medicine, CMSC 2-116, 600 North Wolfe Street, Baltimore, MD 21287 USA

**Keywords:** PEG, Connective tissue disorder, LDS, G-tube, Gastrointestinal, GI, Failure to thrive

## Abstract

**Background:**

Loeys-Dietz syndrome (LDS) is a systemic connective tissue disease (CTD) associated with a predisposition for intestinal inflammation, food allergy, and failure to thrive, often necessitating nutritional supplementation via gastrostomy tube. Poor wound healing has also been observed in in some patients with CTD, potentially increasing the risk of surgical interventions. We undertook to determine the safety and efficacy of gastrostomy tube placement in this population.

**Methods:**

We performed a retrospective cohort study of 10 LDS patients who had a total of 12 gastrostomy tubes placed.

**Results:**

No procedural complications occurred, although one patient developed buried bumper syndrome in the near post-procedural time period and one patient had a small abscess at a surgical stitch. Most patients exhibited improvements in growth, with a median immediate improvement in BMI Z-score of 0.2 per month following the institution of gastrostomy tube feedings. Those with uncontrolled inflammation due to inflammatory bowel disease or eosinophilic gastrointestinal disease showed the least benefit and in some cases failed to demonstrate significant weight gain despite nutritional supplementation.

**Conclusions:**

Gastrostomy tube placement (surgical or endoscopic) is a generally safe and a reasonable therapeutic option for patients with LDS despite their underlying CTD.

## Background

Over 200 disorders have been identified that affect the connective tissue, including a number of genetic disorders that result from single gene mutations. First described in 2005, Loeys–Dietz syndrome (LDS) is an autosomal-dominant connective tissue disorder most commonly caused by mutations in the transforming growth factor β receptor I (*TGFBR1*) and transforming growth factor β receptor II (*TGFBR2*) genes (Type 1 and 2 LDS, respectively) [1, 2]. LDS was initially characterized by aortic aneurysms and generalized arterial tortuosity, hypertelorism, and bifid uvula/cleft palate, although the phenotype was subsequently expanded to include a predilection for the development of food allergies, eosinophilic gastrointestinal disease (EGID), and inflammatory bowel disease (IBD) [3, 4].

Infants and children with LDS frequently present with failure to thrive. The cause is likely multifactorial and may include the impact of repeated surgical interventions and hospitalizations, increased baseline caloric expenditures in patients with inadequately treated asthma and eczema, and unrecognized food allergies and/or intestinal inflammation that result in both increased expenditures and decreased nutrient absorption. Indeed, LDS patients with concomitant food allergy have significantly lower BMI than both normal and children with LDS who do not have food allergy [3]. Additionally, many patients experience difficulty with oral intake due to jaw muscle weakness. Management of nutrition in patients with LDS generally follows traditional protocols. For patients with severe failure to thrive and/or significant allergies, a gastrostomy tube provides stable access. However, while gastrostomy tube placement is generally associated with a low rate of complications in pediatric patients [5, 6], the underlying connective tissue disorder in LDS may pose additional risks which would outweigh the benefits. We therefore undertook a retrospective cohort study of LDS patients treated at our institution to evaluate the safety and efficacy of gastrostomy tube placement in this patient population.

## Methods

This study was approved by the University’s institutional review board. We performed a retrospective cohort study of all patients with a genetically confirmed diagnosis of LDS Types 1 and 2 seen at our institution through December 12, 2017.

## Results

The records of 182 Type 1 and 2 LDS patients were evaluated. Eleven (6%) have undergone gastrostomy tube placement as part of their clinical care. Original records regarding gastrostomy tube placement were not available for one patient who has been excluded from further discussion. Patient characteristics are listed in Table 1. Two of the 10 patients underwent a second gastrostomy tube placement after the originally placed tube was removed (in both cases as a result of patient/family decision). Patient 2 had the second gastrostomy placed endoscopically at the original site. Patient 5 had the second gastrostomy placed at a new site. Half of the patients had Type 1 LDS and half were male. Six patients had allergic or gastrointestinal comorbidities including food allergy, EGID, or IBD. The indication for 10/12 gastrostomy tube placements was failure to thrive. Three patients were less than 1 year of age at the time of placement and had poor oral intake as an indication. Gastrostomy tubes were placed at a median age of 7 years old (yo) and patients were followed for a median of 3.7 years (until the present time or tube discontinuation). Five of 12 placements were done endoscopically, with the remaining being placed surgically by a total of 8 Pediatric Surgeons/Gastroenterologists. The method of placement was at the discretion of the provider.

**Table 1:** Patient Characteristics

Patient	LDS Type	Sex	Comorbidities†	Technique*	age at placement§	Indication	BMI Z-score at placement	BMI ∆ Z-score/ month**	years follow up	Most recent BMI Z-score ‡
1	1	M	FA, EGID, crohns	PEG 14F	10	FTT	−2.32	−0.37	10.4	−6.90
2.1	1	M		Open	< 1	Poor intake	−1.96	−0.04	0.9	
2.2				PEG 20F	3	FTT	−3.63	0.84	5.2	−2.99
3	1	M		Lap w/BIH	< 1	FTT, poor intake	−2.78	0.18	2.1	−1.71
4	1	M		Lap w/LIH	7	FTT	−7.04	0.45	0.7	−3.45
5.1	1	F	FA, eczema	Lap w/Nissen, Ladds for malrotation	8	FTT	−2.10	−0.36	0.7	
5.2				Lap	10	FTT	−4.25	0.02	7.3	−1.97
6	2	M		Lap	9	FTT	−4.25	0.12	8.6	0.34
7	2	F	FA, EGID, UC	PEG 14F	13	FTT	−2.53	−0.25	1.1	−3.96
8	2	F	FA, EGID	PEG 16F	5	FTT	−1.45	−0.03	9.6	−0.46
9	2	F	FA, EGID	Open, Stamm w/Nissen	< 1	Poor intake	−1.56	0.29	13.7	0.54
10	2	F	EGID	PEG 20F	7	FTT	−5.40	0.78	0.6	−0.80
Median					7		−2.66	0.07	6.2	−1.84
Minimum					< 1		−7.04	−0.37	0.6	−6.90
Maximum					13		−1.45	0.84	13.7	0.54

Patients 2 and 5 had gastrostomy tubes placed twice

BMI Z-score from CDC growth chart; at ages less than 2 years old, the WHO weight/length Z-score was used

†*FA* food allergy; *EGID* eosinophilic GI disease; *UC* ulcerative colitis

*PEG Percutaneous endoscopic gastrostomy; *Lap* Laproscopic; *BIH* bilateral inguinal hernia repair; *LIH* Left inguinal hernia repair

§Age at placement rounded to nearest year

**in the immediate post-operative period

‡Most recent BMI Z-score or last BMI Z score prior to discontinuation of gastrostomy tube feeds

**Fig. 1:**
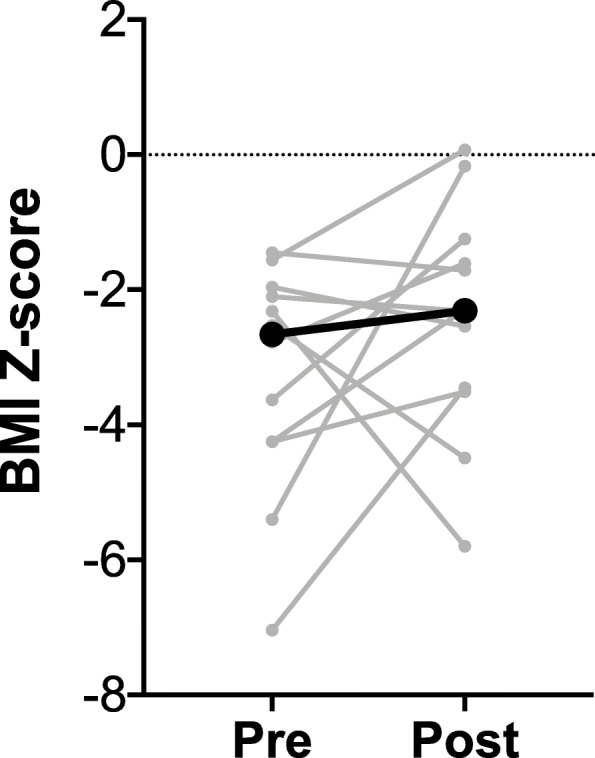
Graphical representation of BMI Z-score change post gastrostomy tube placement (weight/length Z-score change for patients less than 2 years old). Grey lines represent individual patients (including 2 lines for patients 2 and 5 who each had 2 gastrostomy tubes placed). The black line represents the median Z-score change

BMI Z-score based on CDC growth chart (or weight/length Z-score based on WHO growth chart for patients less than 2 years old) was calculated at a physician visit just prior to placement, and again at a physician visit after placement, but at least 2 months post-placement (median 6.7 months post, range 2.8–13.7 months), and are shown in Fig. 1. Median BMI (or weight/length) Z-score prior to placement was − 2.7. Overall, median Z-score change after placement was 0.1 per month. Five patients had negative Z-score changes post-placement. Two of these patients discontinued gastrostomy feeds in the postoperative period prior to the first weight and length measurement (one of these patients had severe vomiting on an intact protein formula, and the tube was removed without a trial of an extensively hydrolyzed or elemental formula; the second patient unilaterally removed the gastrostomy tube halfway through the listed follow-up time). Two patients had severe, uncontrolled IBD. The last patient had EGID and continued eating a food known to trigger her EGID as confirmed by biopsy. Excluding these 5 patients, there was a median 0.3 improvement in Z score per month (range 0.0–0.8) after gastrostomy tube placement. Excluding only the two patients who elected to discontinue feeds through their gastrostomy tube, the Z score increased a median of 0.2 per month (range − 0.4 – 0.8) in the immediate post-procedural time period.

After a median follow-up of 6.2 years (considering only follow-up after the second gastrostomy tube placement for the two patients who received two tubes), all patients had improvement in their BMI Z score over the time period they were receiving feeds through the gastrostomy tube, except for the two patients with IBD. Overall, the BMI Z-score improved a median of 0.2 per year (range − 1.3 – 7.7) for this time period. 60% (7/12) of gastrostomy tube placements resulted in an increase in BMI Z-score in the short term post-operative period. Long-term, 70% (7/10) had improvement in their BMI Z-score.

Evaluation of postoperative complications included infection, bleeding, perforation, or other complication. No procedural complications occurred during the endoscopic or surgical placement of the tubes. Patient 5 had a small abscess at a surgical suture 1 week post placement of her first gastrostomy tube, treated by incision and drainage. Patient 7 had buried bumper syndrome diagnosed 3 weeks after placement. Buried bumper occurs when pressure from the internal bumper leads to migration of the bumper out of the lumen and into the gastric wall along the tract [7]. It is typically considered a late complication of gastrostomy tube placement [8] but has been reported as early as 3 days to 3 weeks post placement [9, 10]. Estimates of the incidence range up to 9% in adults [11] and from 2% to greater than 20% in children [12, 13]. This patient presented with pain after the procedure that progressively worsened. Throughout this time, the outer bumper tension was appropriate, and the tube rotated easily. After 3 weeks a CT scan revealed the buried bumper. A guide wire was placed through the buried PEG tube and visualized in the stomach endoscopically, and the buried tube was removed. The tract was then dilated and a new tube was placed, both over the guidewire. Patient 6 had slow expansion of the gastrostomy site, without signs of gastric prolapse or infection, over months to years that eventually required surgical closure 8.6 years after placement. It is possible the underlying connective tissue disorder contributed to these complications, but we have no direct evidence of this.

## Discussion

Gastrostomy tubes are known to be an effective means to supplement nutrition in children unable to sustain adequate growth via oral intake alone. However, the safety of this procedure in children with an underlying connective tissue disease is not known. Here, we report on the outcome of the placement of 12 gastrostomy tubes in 10 patients with Loeys-Dietz syndrome (LDS), an autosomal dominant connective tissue disease caused by mutations in genes in the TGFβ signaling pathway. LDS is a multisystem disorder associated with an increased propensity for allergic disease, EGID and IBD (both ulcerative colitis and crohns). We have previously demonstrated that the BMI Z-score of patients with LDS are significantly below 0; furthermore, the BMI Z-score of those with comorbid allergic or intestinal disease are significantly below those without these comorbidities [3]. As a result, many of the patients with immune-mediated disease often present with failure to thrive, necessitating supplemental feeding via gastrostomy tube. We found that all 12 tubes placed in this population resulted in no procedural complications. Post-placement, one patient had a small abscess at a surgical stitch and one patient suffered from buried bumper syndrome in the post-operative period, which occurs when excess tension between the internal and external bumpers causes ischemic necrosis of the gastric wall and the tube migrates, becoming lodged between the gastric wall and skin. A third patient had a widely patent/expanded gastrostomy site that required surgical closure. Overall, the procedure was safe in this patient population, whether done endoscopically or surgically.

Among patients who were receiving feeds through their gastrostomy tube, the median BMI (or weight/length for patients less than 2 years old) Z score increased 0.2 per month of gastrostomy tube feedings in the immediate post-procedural time period. The three patients who used their gastrostomy tube but did not have improvement in their BMI Z-score all had ongoing intestinal inflammation (either IBD or EGID), emphasizing that while gastrostomy tubes can provide effective nutritional support, control of underlying inflammatory disease is essential. Long term, all patients using their gastrostomy tube had an increase in their BMI Z-score excepting those with IBD. The selection of formula used post-gastrostomy tube placement should take into account the increased incidence of food allergy and EGID in this population.

## Conclusion

Given the data presented here, gastrostomy tubes can be placed in this population safely. Physicians can recommend placement of a gastrostomy tubes in an LDS patient when clinically indicated.

## Data Availability

The datasets generated and/or analyzed during the current study are not publicly available because they are medical records and therefore protected PHI, but are available from the corresponding author on reasonable request.

## Abbreviations

BIH: Bilateral inguinal hernia repair
BMI: Body mass index
CDC: Centers for Disease Control
CTD: Connective tissue disease
EGID: Eosinophilic gastrointestinal disease
FA: Food Allergy
IBD: Inflammatory bowel disease
Lap: Laproscopic
LIH: Left inguinal hernia repair
LDS: Loeys-Dietz syndrome
PEG: Percutaneous endoscopic gastrostomy
UC: Ulcerative colitis
WHO: World Health Organization
yo: Years old
